# Inhibition of Calcineurin Abrogates While Inhibition of mTOR Promotes Regulatory T Cell Expansion and Graft-Versus-Host Disease Protection by IL-2 in Allogeneic Bone Marrow Transplantation

**DOI:** 10.1371/journal.pone.0092888

**Published:** 2014-03-21

**Authors:** Atsushi Satake, Amanda M. Schmidt, Shosaku Nomura, Taku Kambayashi

**Affiliations:** 1 Department of Pathology and Laboratory Medicine, Perelman School of Medicine at the University of Pennsylvania, Philadelphia, Pennsylvania, United States of America; 2 First Department of Internal Medicine, Kansai Medical University, Osaka, Japan; Maisonneuve-Rosemont Hospital, Canada

## Abstract

Regulatory T cells (Treg)s attenuate excessive immune responses, making their expansion beneficial in immune-mediated diseases including allogeneic bone marrow transplantation (BMT)-associated graft-versus-host disease (GVHD). We have recently reported that Treg expansion does not require phospholipase Cγ activation when IL-2 is provided. As such, the combination of IL-2 and a calcineurin inhibitor (Cyclosporine A; CsA) expands Tregs while inhibiting Tconv proliferation and protects against a mouse model of multiple sclerosis. However, CsA inhibits Treg proliferation in the presence of a TCR stimulus, suggesting that CsA may negatively impact Treg proliferation when they receive strong allogeneic MHC-mediated TCR signals. In this study, we show that CsA inhibits Treg proliferation and inducible Treg generation in allogeneic but not in syngeneic BMT when IL-2 is provided. In contrast to CsA, the mTOR inhibitor (Rapamycin) almost completely suppressed IL-2-mediated Treg proliferation. However, CsA and Rapamycin inhibited Treg proliferation to a similar extent when TCR stimulation was provided. Furthermore, Rapamycin promoted Treg expansion and inducible Treg generation in allogeneic BMT recipients treated with IL-2. Consistent with these observations, CsA abrogated while Rapamycin promoted the protective effect of IL-2 on allogeneic BMT-induced GVHD. These results suggest that while CsA permits IL-2-induced Treg proliferation in the syngeneic setting (absence of strong TCR signals), CsA in combination with IL-2 may be detrimental for Treg proliferation in an allogeneic setting. Thus, in allogeneic settings, an mTOR inhibitor such as Rapamycin is a better choice for adjunct therapy with IL-2 in expansion of Tregs and protection against allogeneic BMT-induced GVHD.

## Introduction

To maintain immune tolerance, pathogenic self MHC-reactive T cells are excluded by negative selection in the thymic medulla. Nevertheless, some T cells that emigrate to the periphery still have an ability to mount autoimmune responses. To attenuate the response of such self-reactive T cells and to limit immunopathology in overexuberant immune responses directed against foreign antigens, several peripheral tolerance mechanisms are in place. One important process involves the inhibition of conventional T cells (Tconv)s by regulatory T cells (Treg)s, a subset of T cells with suppressive properties [1], [2]. Patients and mice with mutations of the Treg lineage-determining transcription factor, Foxp3, harbor no Tregs and display Tconv hyperreactivity [3]. As such, they succumb to lethal systemic autoimmunity unless transplanted with allogeneic hematopoietic stem cells that reconstitute their immune system with functional Tregs. In addition to limiting T cell responses against self MHC/peptide complexes and to pathogens, Tregs also prevent allogeneic T cell responses observed in graft rejection and graft-versus-host disease (GVHD), a frequent and severe complication in hematopoietic stem cell transplantation [4]–[6]. Therefore, the selective enrichment of Tregs is a promising strategy to regulate harmful immune responses against allogeneic antigens.

A deep understanding of the mechanisms that regulate Treg proliferation is necessary to devise strategies for the selective expansion of Tregs. For optimal proliferation, Tregs require T cell receptor (TCR) and interleukin (IL)-2 signaling for their homeostasis and proliferation in the periphery [7]–[9]. However, we and others have shown that Tregs can proliferate in a TCR-independent manner if exogenous IL-2 is provided [10], [11]. More specifically, we found that phospholipase Cγ (PLC)γ activation is not required for IL-2-induced Treg proliferation. Because Tconvs require PLCγ activation for their proliferation, we hypothesized that a combination of IL-2 and pharmacological TCR inhibition downstream of PLCγ will expand Tregs *in vivo* while suppressing Tconv proliferation. Indeed, treatment of mice with a calcineurin inhibitor (cyclosporine A; CsA) and IL-2 led to an increase in Tregs and a decrease in antigen-specific T cell expansion, resulting in attenuated disease severity in experimental autoimmune encephalomyelitis [12]. However, CsA inhibited Treg proliferation in the presence of a TCR stimulus, suggesting that CsA may negatively impact Treg proliferation when they receive strong allogeneic MHC-mediated TCR signals.

To test this notion, we hereby investigated the impact of pharmacological TCR signaling inhibition and IL-2 on the expansion of Tregs in the allogeneic setting. Using a mouse bone marrow transplantation (BMT) model, we show that the combination of CsA and IL-2 expands Tregs in syngeneic BMT but inhibits Treg expansion and inducible Treg (iTreg) generation in allogeneic BMT. In contrast, Rapamycin (Rapa), an mTOR inhibitor, promoted Treg expansion and inducible Treg (iTreg) generation in allogeneic BMT. Consistent with these observations, we found that CsA abrogates while Rapa promotes the protective effect of IL-2 on GVHD in allogeneic BMT. These results suggest that while CsA permits IL-2-induced Treg proliferation in the syngeneic setting (absence of strong TCR signals), CsA in combination with IL-2 may be detrimental for Treg proliferation in an allogeneic setting. Thus, in allogeneic settings, an mTOR inhibitor such as Rapa is a better choice for adjunct therapy with IL-2 in expansion of Tregs and protection against allogeneic BMT-induced GVHD.

## Materials and Methods

### Mice

C57BL/6 (B6), B6D2F1, and B6.SJL (CD45.1 congenic) mice were purchased from the National Cancer Institute. Foxp3 green fluorescent protein knock-in (Foxp3.GFP KI), mice were purchased from Jackson Laboratories. B6D2F1 (CD45.1/CD45.2 heterozygous) and CD45.1^+^ Foxp3.GFP KI mice were created by crossing B6.SJL mice to DBA2 mice and Foxp3.GFP KI mice, respectively. Mice were 6 to 16 weeks of age at time of sacrifice. Mice were housed in specific pathogen-free conditions and treated in strict compliance with Institutional Animal Care and Use Committee (IACUC) regulations of the University of Pennsylvania. All animal studies were approved by the IACUC, protocol number 804245.

### Flow cytometry, cell sorting, and data analysis

Antibodies for flow cytometry were purchased from BD Pharmingen (San Diego, CA): Fc block (2.4G2), anti-CD25 (PC61), anti–CD4 (RM4-5); eBioscience (San Diego, CA): anti-Foxp3 (FJK-16s), anti-CD45.1 (A20); Biolegend (San Diego, CA): anti-CD45.1 (A20), anti-CD45.2 (104), anti-CD8α (53–6.7), anti-CD3 (17A2); Molecular Probes, Invitrogen (Carlsbad, CA): LIVE/DEAD Fixable Aqua Dead Cell Stain Kit and CFSE. Cells were stained as previously reported and analyzed by an LSR II or a FACSCanto (BD Biosciences, San Jose, CA). For cell sorting, T cells were purified with CD4 and CD8 magnetic beads using MACS columns (Miltenyi Biotec, Auburn, CA) prior to cell surface staining. FACS sort was performed with a FACSAria cell sorter (BD Biosciences) at the University of Pennsylvania Flow Cytometry and Cell Sorting Core. FACS-sorted populations were typically of >95% purity. Data were analyzed with FlowJo software (TreeStar, Ashland, OR). Dead cells were excluded from analysis with LIVE/DEAD Fixable Aqua Dead Cell staining. Statistical analysis was performed by t test, paired t test, ANOVA, or log-rank test using Prism (GraphPad) as appropriate.

### *In vitro* Treg proliferation assays

For Treg proliferation assays, FACS-sorted Foxp3.GFP^+^CD4^+^ Tregs were labeled with CFSE. CFSE-labeled Tregs (1×10^4^ cells/well) and MACS-sorted dendritic cells (DC)s (1×10^5^ cells/well) were plated in 200 μl T cell media (MEM-α with 10% FBS, 1% penicillin/streptomycin, 10 mM HEPES, and 1×10^−5^ M 2-mercaptoethanol) with mouse granulocyte macrophage colony stimulating factor (10 ng/ml; PeproTech, Rocky Hill, NJ) and human IL-2 (50 U/ml; PeproTech) in 96-well flat bottom plates. CD11c^+^ DCs were obtained from spleens of mice subcutaneously injected 8–10 days prior with FLT3L-expressing EL4 cells. Cells were cultured with or without the indicated factors at 37°C and analyzed by flow cytometry 4 days later.

### Induction and assessment of GVHD

B6D2F1 mice were irradiated with a total of 1000 cGy in 2 equal doses separated by 12 hours. Irradiated mice were intravenously injected with 5×10^6^ spleen cells and 3×10^6^ T-cell-depleted BM cells from B6 mice. For detection of Treg subsets during GVHD, irradiated B6D2F1 mice were injected with 1.5×10^6^ FACS-sorted Tconvs (CD45.1^+^CD4^+^Foxp3.GFP^−^ and CD45.1^+^CD8^+^Foxp3.GFP^−^), 0.15×10^6^ FACS-sorted Tregs (CD45.2^+^CD4^+^Foxp3.GFP^+^), and 3×10^6^ T cell–depleted BM cells (CD45.2^+^). Host mice were treated with vehicle (PBS), CsA (25 mg/kg), or Rapa (0.5 mg/kg) for 5 days (days 0–4) with or without concomitant IL-2 immune complexes (IL-2 IC)s (1.5 μg/mouse) for 3 days (days 0–2). IL-2 ICs were prepared by mixing 1.25 μg of anti-IL-2 antibody (clone JES6-1D; BioXCell, West Lebanon, NH) with 0.25 μg of mouse IL-2 (eBioscience) for 30 minutes on ice in 200 μl PBS. PBS, CsA, Rapa and IL-2 ICs were injected intraperitoneally as indicated. Mice were monitored every day for survival. The degree of clinical GVHD was assessed 2–3 times per week by a scoring system that sums changes in 5 clinical parameters: wt loss, posture, activity, fur texture, and skin integrity [13]. Mice were euthanized when their wt dropped to <30% of their initial body wt.

### Treg suppression assays

1×10^6^ MACS-enriched CD4^+^ T cells from Foxp3.GFP reporter mice (CD45.2^+^) were cultured in 6-well tissue culture plates with 5×10^6^ irradiated splenocytes (feeder cells) with anti-CD3/CD28 (1 μg/ml each) and IL-2 in the presence or absence of Rapa (10 ng/ml) or CsA (100 ng/ml) for 5 days. The expanded GFP^+^ Tregs, freshly isolated GFP^+^ Tregs from Foxp3.GFP reporter mice, and CD4^+^Foxp3^−^ Tconvs from WT B6.SJL Foxp3.GFP reporter mice (CD45.1^+^) were FACS-sorted. The CD4^+^Foxp3^−^ Tconvs were CFSE-labeled and 1.5×10^4^ CFSE-labeled Tconvs were cultured at various ratios with Tregs (starting at 1.5×10^4^ cells) in the presence of irradiated T cell-depleted CD45.2^+^ feeder cells (1.5×10^5^ cells) and soluble anti-CD3 (1 μg/ml) in 96-well flat bottom tissue culture plates. CFSE dilution of Tconvs (CD4^+^CD45.1^+^) was assessed by flow cytometry after 4 days in culture and the division index of Tconvs was assessed using FlowJo software. The division index represents the average number of cell divisions occurring for the gated population.

### *In vitro* iTreg conversion assay

5×10^4^ FACS-sorted Tconvs (CD4^+^Foxp3.GFP^−^) and CD8^+^ T-cells (CD8^+^Foxp3.GFP^−^) from Foxp3.GFP-reporter mice were stimulated in 96-well culture plates pre-coated with anti-CD3 (2 μg/mL) and anti-CD28 (2 μg/mL) in the presence of IL-2 (50 U/ml) and human TGFβ (0.2 ng/ml for CD4^+^ and 1 ng/ml for CD8^+^ T-cell cultures). In some wells, CsA (100 ng/ml) and/or Rapa (10 ng/ml) were added. Five days later, the T cells were analyzed for Foxp3 expression by flow cytometry.

## Results

### Rapa but not CsA inhibits IL-2-induced Treg proliferation

**Figure 1:**
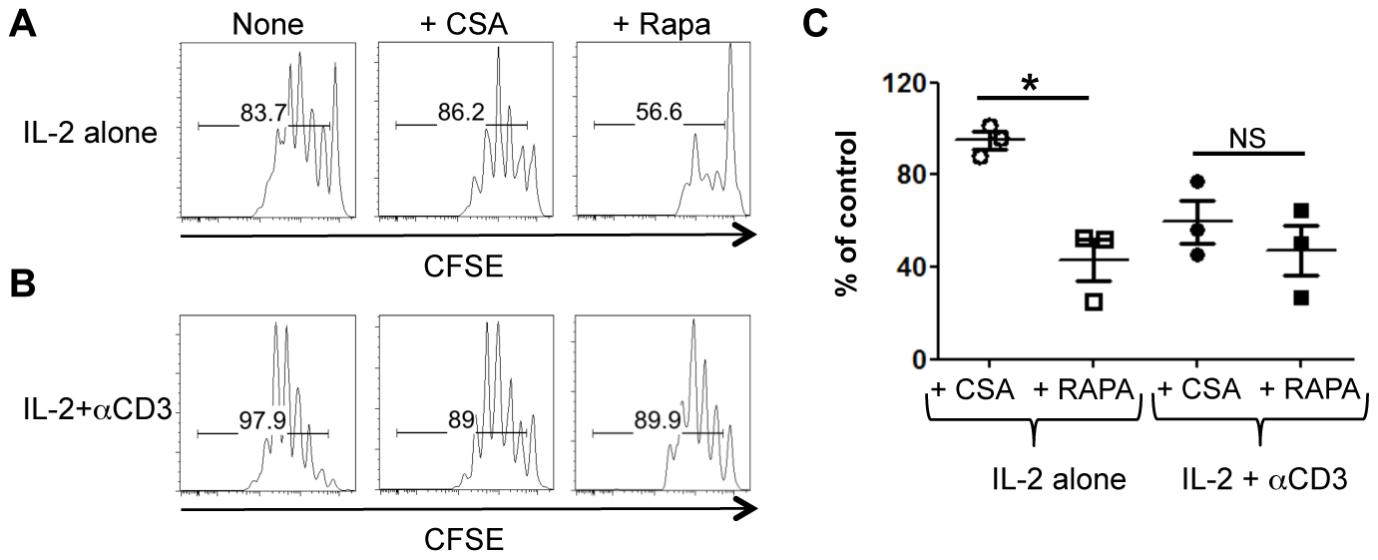
Calcineurin signaling restores Treg proliferation in the presence of Rapa. CFSE-labeled FACS-sorted Tregs were co-cultured with B6-derived DCs and IL-2 with or without Rapa or CsA in the absence (A) or presence (B) of anti-CD3 antibody. One representative histogram showing Treg CFSE dilution in each condition is shown. (C) The division index of Tregs cultured in the presence of anti-CD3 antibody was normalized to the control and is plotted as mean ± SEM of *n* = 3 independent experiments. *p<0.01 by unpaired, two-tailed Student t test.

Treg proliferation *in vitro* requires IL-2 and interactions with dendritic cells (DC)s. In this setting, co-stimulatory molecules (CD80, CD86, and OX40L) but not MHC class II expression by DCs are required for IL-2-induced Treg proliferation [10], [11]. Consistent with this observation, we recently reported that CsA minimally affects Treg proliferation induced by IL-2 and DCs [12]. Since the AKT-mTOR pathway is a major signaling component downstream of co-stimulatory signals, we tested how the mTOR inhibitor (Rapa) would affect Treg proliferation induced by IL-2 and DCs. As predicted, Rapa but not CsA inhibited the proliferation of Tregs induced by IL-2 and DCs (Fig. 1A), highlighting the importance of costimulatory signals in this setting.

### Rapa and CsA inhibit IL-2-induced Treg proliferation to a similar extent in the presence of overt TCR stimulation

We previously reported that CsA inhibits Treg proliferation when stimulated with anti-CD3, IL-2, and DCs, suggesting that calcineurin activation is crucial for IL-2-induced Treg proliferation in the presence of TCR stimulation. To test how this might compare to mTOR inhibition by Rapa, we tested the effects of Rapa and CsA on Treg proliferation induced by IL-2 and DCs in the presence of anti-CD3. Treg proliferation was suppressed by CsA in the presence of anti-CD3, such that CSA and Rapa now equally inhibited Treg proliferation in this setting (Figure 1A, B, and C). These data suggest that in the presence of TCR stimulation, both calcineurin and mTOR signaling is required for the optimal proliferation of Tregs.

### IL-2 plus CsA suppresses while IL-2 plus Rapa promotes the expansion of pre-existing donor-derived Treg expansion and inducible Treg (iTreg) generation during allogeneic BMT

**Figure 2:**
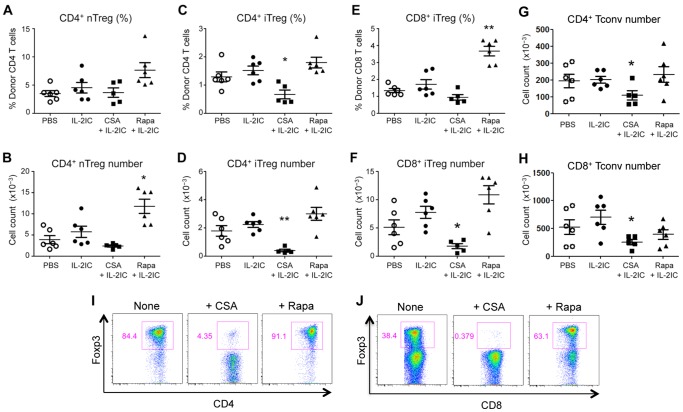
CsA suppresses while Rapa promotes pre-existing donor-derived Treg expansion and iTreg generation during GVHD. GFP^−^ Tconvs (CD4^+^ and CD8^+^) and GFP^+^CD4^+^ Tregs were FACS-sorted from CD45.1^+^ Foxp3 GFP-reporter mice and CD45.2^+^ Foxp3 GFP-reporter mice, respectively, combined with T cell-depleted BM cells (CD45.2^+^), and injected into irradiated B6D2F1 mice. Host mice were treated with vehicle (PBS), IL-2 ICs, IL-2 ICs plus CsA (25 mg/kg), or IL-2 ICs plus Rapa (0.5 mg/kg) for 5 days. IL-2 ICs were given only for the first 3 days (Days 0, 1, and 2). Eight days after BMT, donor Tregs in the spleen were analyzed by flow cytometry. (A) The percentage and (B) absolute number of pre-existing donor nTregs, (C) the percentage and (D) absolute number of CD4^+^ iTregs, (E) the percentage and (F) absolute number of CD8^+^ iTregs, (G) the absolute number of CD4^+^ Tconvs, and (H) the absolute number of CD8^+^ Tconvs are plotted as mean ± SEM of *n* = 5–6 mice/group from two independent experiments. *p<0.05; **p<0.001 by two-tailed Student t test compared with the group treated with IL-2 ICs. (I) FACS-sorted CD4^+^ or (J) CD8^+^ Foxp3.GFP^−^ Tconvs were stimulated with plate-bound anti-CD3/anti-CD28 and soluble TGFβ and IL-2 with or without CsA (100 ng/ml) or Rapa (10 ng/ml). 4 days later, the cells were harvested and analyzed by flow cytometry. One representative plot from 2 independent experiments is shown.

**Figure 3:**
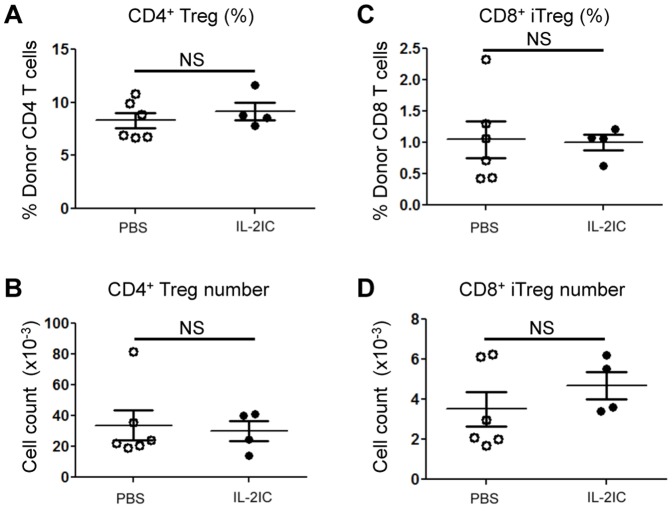
IL-2 ICs do not expand CD4^+^ or CD8^+^ Tregs on Day 5 post allogeneic BMT. Spleen cells and T-cell-depleted BM cells from WT B6.SJL mice (CD45.1^+^) were injected into irradiated B6 (CD45.2^+^) mice. Host mice were treated with vehicle (PBS) or IL-2 ICs for 3 days (Days 0, 1, and 2). Five days after BMT, donor CD4^+^ and CD8^+^ Tregs in the spleen were analyzed by flow cytometry. (A) The percentage and (B) absolute number of CD4^+^ Tregs and (C) the percentage (E) and absolute number (F) of CD8^+^ iTregs are plotted as mean ± SEM of *n* = 4–6 mice/group from two independent experiments. NS  =  not significant by two-tailed Student t test.

To test how CsA and Rapa impact Treg proliferation in the setting where Tregs receive strong TCR signals *in vivo*, we employed an allogeneic BMT model and examined the Treg pool in allogeneic BMT mice administered IL-2 (in the form of immune complexes; IL-2 ICs) with or without CsA or Rapa. During allogeneic BMT, the pool of Tregs is formed by expansion of pre-existing donor-derived Tregs and by the conversion of donor-derived Tconvs into iTregs in the recipient. We examined both subsets of Tregs, since a large fraction of the Treg pool during GVHD consists of CD4^+^ and CD8^+^ iTregs that have converted from alloreactive naïve Tconvs and contribute to GVHD protection. [14]–[16]. The different subsets of Tregs were distinguished by mixing FACS-sorted congenically disparate (CD45.1 vs. CD45.2) allogeneic Tregs and Tconvs (CD4^+^ and CD8^+^) and injecting them together with the BM cells into the irradiated recipients. Consistent with our *in vitro* results, CsA plus IL-2 treatment reduced the total number of pre-existing donor-derived Tregs compared to treatment with IL-2 alone on Day 8 post-BMT (Figure 2A and B). In contrast, Rapa augmented Treg numbers when given in conjunction with IL-2 (Figure 2A and B). Moreover, the absolute number and percentages of CD4^+^ and CD8^+^ iTregs were markedly decreased in CSA plus IL-2-treated mice but enhanced in Rapa plus IL-2-treated mice (Figure 2C–F). The relative preservation of Treg percentages in CSA plus IL-2-treated mice could be due to the reduction of Tconv numbers in these mice (Figure 2G–H). Although RAPA plus IL-2 treatment slightly but not significantly decreased CD8^+^ Tconv numbers, no effect on CD4^+^ Tconv numbers was observed. A similar effect of Rapa and CsA was observed with *in vitro* CD4^+^ and CD8^+^ iTreg conversion assays, whereby Rapa augmented and CsA suppressed iTreg formation (Figure 2I–J). Notably and unexpectedly, IL-2 treatment did not significantly increase the total numbers or percentages of CD4^+^ and CD8^+^ Tregs at Day 8 post BMT. This was also true at an earlier time point (Day 5 post BMT; Figure 3), suggesting that IL-2 treatment does not increase Treg numbers in the allogeneic BMT setting at these time points.

**Figure 4:**
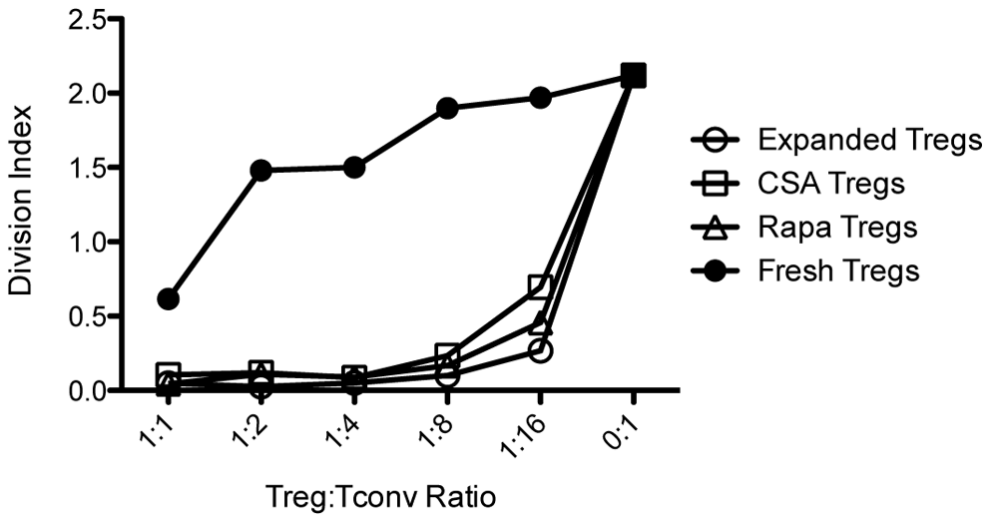
IL-2 plus anti-CD3-expanded Tregs in the presence or absence of CsA or Rapa suppress Tconv proliferation. MACS-sorted CD4^+^ T cells from CD45.2^+^ Foxp3 GFP-reporter mice were cultured with irradiated feeder cells in IL-2 plus anti-CD3/CD28 in the presence or absence of CsA or Rapa. 5 days later, GFP^+^ Tregs were sorted by flow cytometry and co-cultured with CFSE-labeled FACS-sorted splenic Foxp3^−^CD4^+^ T cells (Tconvs) from CD45.1^+^ Foxp3 GFP-reporter mice at various Tconv:Treg ratios for four days in the presence of irradiated T cell-depleted splenocytes and anti-CD3. FACS-sorted GFP^+^ Tregs from freshly isolated splenocytes of CD45.2^+^ Foxp3 GFP-reporter mice were also used for comparison. The division index of Tconvs is plotted against various Treg:Tconv ratios. One representative of *n* = 2 independent experiments is shown.

To test the impact of CsA and Rapa on Treg function, we next tested the suppressive ability of Tregs that were expanded in IL-2/anti-CD3 in the presence or absence of CsA or Rapa. Compared to freshly isolated Tregs, IL-2/anti-CD3-expanded Tregs showed markedly increased inhibitory ability against Tconv proliferation (Fig. 4). This was true even when the Tregs were expanded in CsA or Rapa.

**Figure 5:**
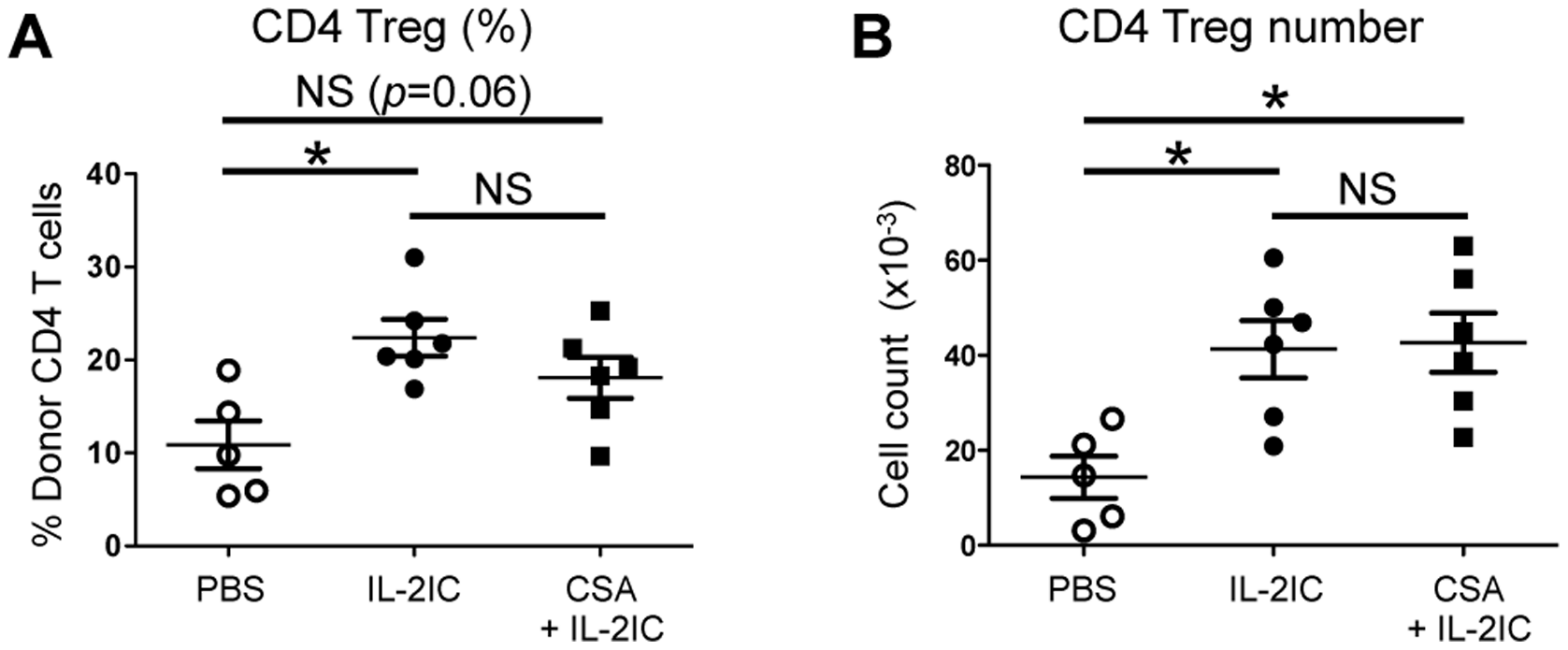
CsA does not inhibit IL-2-induced Treg expansion in the absence of overt TCR stimulation. Spleen cells and T-cell-depleted BM cells from WT B6.SJL mice (CD45.1^+^) were injected into irradiated B6 (CD45.2^+^) mice. Host mice were treated with PBS alone, IL-2 ICs alone, or CsA + IL-2 ICs as well as the GVHD model. Eight days after BMT, donor CD4^+^Tregs (CD45.1^+^) in the spleen analyzed by flow cytometry. (A) The percentage and (B) absolute number of donor CD4^+^Tregs are plotted as mean ± SEM of *n* = 5–6 mice/group from two independent experiments. *p<0.05 by two-tailed Student t test compared with the group treated with IL-2 ICs.

Although our data suggested that CsA suppresses and Rapa augments Treg numbers in allogeneic BMT in the presence of IL-2, we could not exclude the possibility that the inhibitory effect of CsA on Treg expansion was secondary to inflammation and lymphopenia induced by myeloablative conditioning (irradiation) during BMT. To test this possibility, IL-2 and CsA were administered to mice receiving syngeneic BMT with the same amount of irradiation. In contrast to the allogeneic BMT setting, IL-2 treatment resulted in an expansion of Tregs, which was not inhibited by CsA (Figure 5A–B). Together, these data suggest that CsA blocks nTreg proliferation and iTreg generation in allogeneic BMT mice treated with IL-2.

### CsA blocks while Rapa augments the protective effect of IL-2 in GVHD

**Figure 6:**
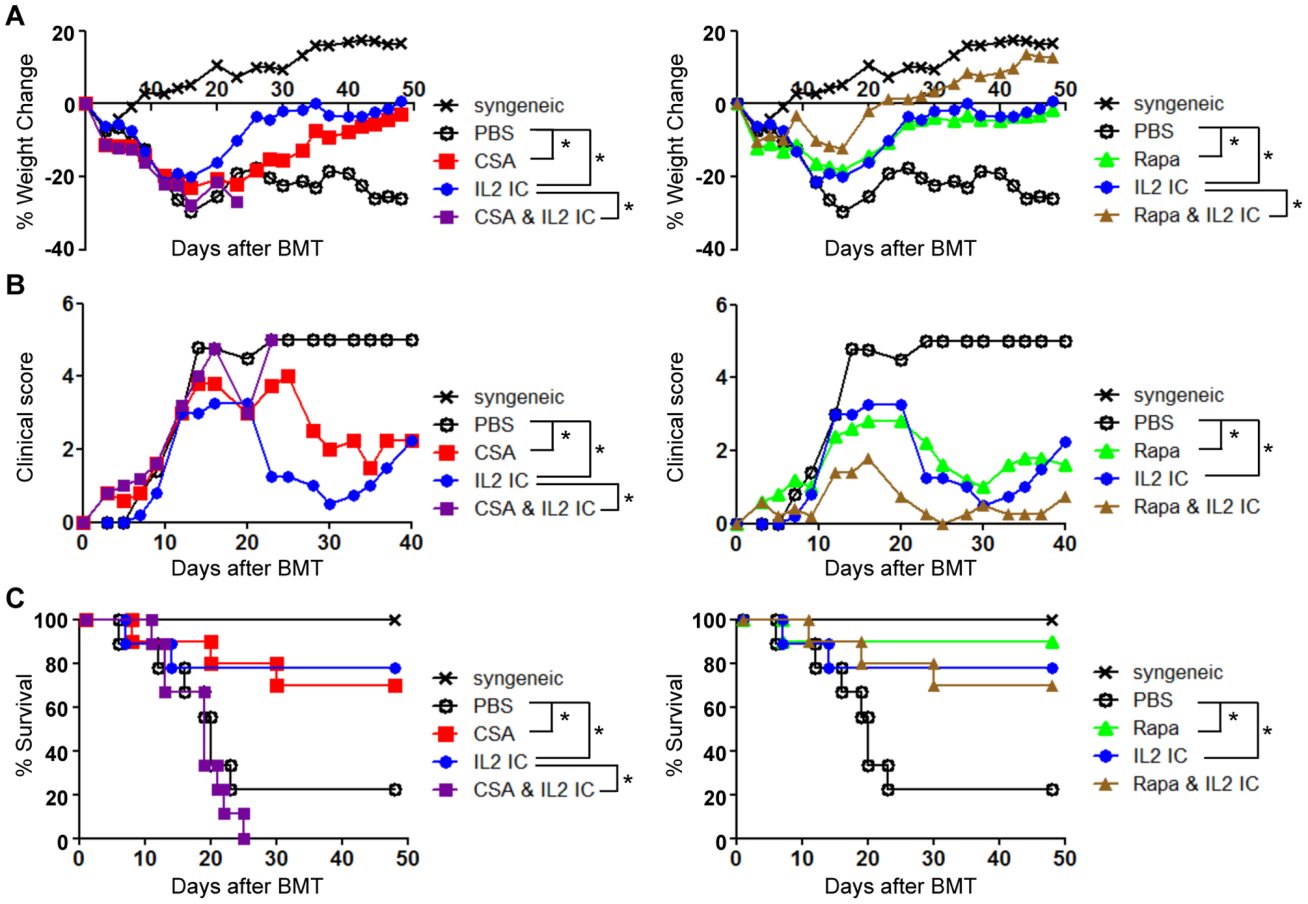
CsA abolishes while Rapa promotes the beneficial effect of IL-2 ICs in protection against acute GVHD. B6 splenocytes and T cell-depleted BM cells were injected into irradiated B6D2F1 mice. The mice were treated with either vehicle (PBS), CsA, Rapa, IL-2 ICs, CsA + IL-2 ICs, or Rapa + IL-2 ICs on days 1–3. Changes in (A) wt and (B) GVHD score are plotted as the mean of *n* = 5 mice/group vs. days post transplant. One representative of 2 independent experiments is shown. *p<0.05 by ANOVA with Tukey's post hoc test. (C) Survival was monitored over a 7-week period. Two independent experiments were combined with a total of *n* = 9 or 10 mice/group to generate the survival curve. *p<0.05 by log-rank test. Note that the CsA and Rapa treatment groups were separated into two graphs (left and right) for visualization purposes. The syngeneic and PBS-treated groups are identical for both plots.

To test whether the effects of CsA and Rapa on Treg numbers correlated with GVHD protection, allogeneic BMT mice were treated with various combinations of IL-2, Rapa, and CsA and monitored for weight loss, GVHD clinical score, and mortality. The injection of IL-2 to allogeneic BMT mice significantly protected against GVHD-induced weight loss, clinical score, and mortality. Furthermore, as reported recently by others [17], Rapa and IL-2 displayed synergism in protecting against GVHD-induced weight loss (Figure 6A), although we could not observe a significant synergistic effect in clinical score or GVHD mortality (Figure 6B-C). Although CsA alone demonstrated significant benefit in protection against GVHD, the combination of CsA and IL-2 severely and significantly worsened disease outcome including weight loss, clinical score, and mortality (Figure 6A–C). These results suggest that the combination of CsA and IL-2 is detrimental in treatment of acute GVHD, which correlates with the negative effects of CsA on Tregs in the allogeneic BMT setting.

## Discussion

Understanding the requirements for Treg proliferation is important to devise strategies involving the manipulation of Treg numbers in various disease settings. Previous studies from our laboratory and others demonstrated that Treg expansion does not rely heavily on TCR stimulation as long as exogenous IL-2 is provided [11], [12]. As such, pharmacological inhibition of the TCR signaling pathway by CsA had little impact on IL-2-induced Treg proliferation *in vitro* and *in vivo* in a syngeneic setting. However, when additional TCR stimulation was provided in an *in vitro* assay, the proliferation of Tregs was markedly enhanced and now became an important signal for maximal Treg proliferation [12]. This led us to hypothesize that the requirements of Treg proliferation could be different in an allogeneic BMT setting, where a fraction of the Tregs would be expected to receive strong allogeneic MHC-driven TCR signals. To test this notion, we examined the impact of CsA on Tregs in an allogeneic BMT setting and found that CsA negatively affected Treg numbers and iTreg generation even in the presence of IL-2. This negative effect of CsA correlated with a significantly exacerbated GVHD outcome in mice treated with CsA and IL-2. In contrast, CsA did not affect Treg numbers in syngeneic BMT mice treated with IL-2.

In addition to investigating the effects of CsA on Treg numbers, we examined the role of the mTOR signaling pathway under the same conditions. In *in vitro* assays, we previously found that costimulatory signals provided by DCs was critical for Treg proliferation in the absence of MHC class II [18]. Thus, we predicted that the mTOR inhibitor Rapa would suppress Treg proliferation, as AKT-mTOR is a critical signaling pathway downstream of costimulatory molecules. Indeed, Rapa inhibited Treg proliferation induced by IL-2 and DCs. However, in contrast to CsA treatment, Rapa did not reduce the Treg proliferation further when TCR stimulation was provided. In fact, in an allogeneic BMT setting, IL-2 and Rapa synergized to increase Treg numbers and enhance iTreg generation. The increase in iTreg generation is consistent with results from our *in vitro* iTreg conversion assays, where Rapa augmented IL-2/TGFβ-induced conversion of Tconvs into iTregs. However, why pre-existing nTregs were also expanded by IL-2 and Rapa in allogeneic BMT is unclear, as Rapa partially inhibited Treg proliferation *in vitro* even in the presence of anti-CD3. One possibility is that Rapa has a strong negative effect on Tconvs [19], [20], leading to an increased Treg:Tconv ratio that allowed IL-2 to expand Tregs more efficiently. However, our data suggest that only CD8^+^ Tconvs and not CD4^+^ Tconvs are affected by Rapa plus IL-2 treatment. Thus, the discrepancy between the *in vitro* and *in vivo* effect of Rapa on Treg numbers and the Treg:Tconv ratio in the allogeneic BMT setting cannot be explained at this time and warrants further investigation.

GVHD-induced weight loss but not survival or clinical score was improved with the combination of Rapa and IL-2 compared to either treatment alone. Compared to previous findings described by Shin et al. [17], the synergistic effects of IL-2 and Rapa on GVHD protection were not as pronounced in our study. This could potentially be due to differences in the GVHD induction protocol. While Shin et al. used purified Tconvs to induce GVHD, our study was performed with splenocytes that contain both Tconvs and Tregs. Thus, the strong effect of Rapa in augmenting the IL-2-mediated conversion of Tconvs into iTregs could have contributed more to protection against GVHD in the study by Shin et al. Nevertheless, even in the presence of nTregs, our data support a beneficial effect in combining Rapa and IL-2 in the treatment of GVHD.

The combination of CsA and IL-2 is highly effective against EAE compared to treatment with either agent alone [12]. This is in striking contrast to the acute GVHD setting, where CsA suppressed the beneficial effect of IL-2 on disease outcome. We speculate that these differences arise, because strong allogeneic MHC-stimulated TCR signals drive nTreg proliferation to keep up with the expansion of alloreactive Tconvs. Thus, as opposed to the syngeneic setting, TCR signals become critical in nTreg expansion, which is inhibited by CsA. In addition, the negative effect of CsA on iTreg generation may also contribute to the detrimental effect of CsA, as both CD4^+^ and CD8^+^ iTregs are important in GVHD protection [14]–[16]. However, other differences in EAE compared to GVHD including the precursor frequency of the disease-specific T-cells (high in GVHD), the fullness of the T-cell compartment (lymphopenia in GVHD), and the degree of inflammation (high in GVHD) also need to be considered.

Another intriguing observation is that CsA negatively impacted GVHD outcome when given with IL-2 but was protective when given alone. IL-2 is a crucial factor for Treg survival and growth [21], [22] and can be utilized by Tregs more effectively than Tconvs because of their constitutive expression of the high affinity IL-2 receptor CD25/CD122/CD132 complex. While this is true in the absence of inflammation, Tconvs inducibly express the high affinity IL-2 receptor complex upon activation. Thus, during massive T cell activation occurring during GVHD, excessive IL-2 may expand not only Tregs but also Tconvs. In the present study, we limited the injection of IL-2 to the first 3 days, as we observed high mortality of hosts in which IL-2 was administered continuously (data not shown). Mice injected continuously with IL-2 showed an expansion of highly activated Tconvs, suggesting that extra IL-2 would augment pathogenic Tconvs harmful to the allogeneic BMT host. Thus, the negative effects of IL-2 may be brought out by the concurrent suppression of Tregs by CsA, which would allow IL-2-mediated expansion and activation of Tconvs. Alternatively, the mechanism of action of CsA could be in part mediated by suppression of IL-2 production from alloreactive Tconvs, which is necessary for their expansion. If fact, the reduction in IL-2 production by CsA treatment alone has been proposed to negatively affect Treg numbers during GVHD [23]. Although the provision of IL-2 in combination with CsA did not rescue the Treg defect during allogeneic BMT in our study, it could still potentially reverse the beneficial inhibitory effects that CsA has on suppressing alloreactive Tconv proliferation. While we do not know the exact mechanism of why CSA and IL-2 cancel out each other's beneficial effects in the treatment of acute GVHD, we propose that this combination should be avoided in this disease setting.

The failure of IL-2 treatment to increase absolute numbers of Tregs during allogeneic BMT was unexpected. This may occur because the Tregs in the allogeneic setting are maximally proliferating such that the expansion cannot be further augmented. Indeed, previous studies have shown that Treg proliferation is more dependent on TCR stimulation than on IL-2 in a lymphopenic setting [7]. This then begs the question of why IL-2 treatment attenuates GVHD in the absence of Treg expansion. It is possible that IL-2 protects against GVHD by augmenting Treg function. In our previous study [12], we found that Tregs isolated from IL-2-treated mice are significantly more suppressive than Tregs from PBS-treated mice.

Alterations in Treg function could also partially contribute to the negative effects of CsA in IL-2-mediated protection against GVHD, as Treg function was slightly reduced when Tregs were expanded with anti-CD3 and IL-2 in the presence of CsA. In addition to its effects on Treg proliferation, NFAT, the transcription factor downstream of calcineurin, plays a critical role for the ability of Tregs to exert their suppressive function [24]. Thus, many factors contribute to why a calcineurin inhibitor would not synergize with IL-2 to protect against acute GVHD. Although the combination of calcineurin inhibition and IL-2 might be detrimental in acute GVHD as shown by our current study, it could be still effective in chronic GVHD. In contrast to acute GVHD that is caused by alloreactive mature T cells, chronic GVHD is potentially caused by host thymic dysfunction leading to inadequate negative selection and/or Treg generation from the thymus [25], [26]. Chronic GVHD presents with autoimmune disease manifestations including scleroderma, lupus, and Sjogren-like conditions. Thus, chronic GVHD may mimic more of a syngeneic autoimmune disease scenario, where CsA and IL-2 combination therapy might be beneficial. In support of this notion, IL-2 treatment was found to be effective against chronic GVHD in humans and although more than 50% of patients were concomitantly treated with a calcineurin inhibitor in this clinical study, many of them still exhibited an elevation in Treg numbers and showed disease improvement [27].

In summary, we have demonstrated that pharmacological TCR inhibition plus IL-2 does not allow the expansion of nTregs and the generation of iTregs in the allogeneic BMT setting. Unlike the syngeneic setting, CsA and IL-2 does not suppress allogeneic immune responses, indicating that IL-2 treatment against acute GVHD would not be recommended in the clinical setting because almost all recipients are treated with a calcineurin inhibitor for GVHD prophylaxis. Thus, depending on the setting, the signaling pathways leading to expansion of the Treg pool need to be carefully considered and the immunosuppressive regimen needs to be tailored to most effectively increase the Treg:Tconv ratio.
